# Structural and functional characterization of *M. tuberculosis* sedoheptulose- 7-phosphate isomerase, a critical enzyme involved in lipopolysaccharide biosynthetic pathway

**DOI:** 10.1038/s41598-020-77230-8

**Published:** 2020-11-30

**Authors:** Sumita Karan, Bhanu Pratap, Shiv Pratap Yadav, FNU Ashish, Ajay K. Saxena

**Affiliations:** 1 Rm-403/440, Structural Biology Lab, School of Life Sciences, Jawaharlal Nehru University, New Delhi, 110067 India; 2 Protein Science and Engineering Division, Institute of Microbial Technology, Sector 39-A, Chandigarh, 160036 India

**Keywords:** SAXS, Biological physics

## Abstract

*M. tuberculosis* GmhA enzyme catalyzes the isomerization of D-sedoheptulose 7-phosphate into D-glycero-D-α-manno-heptose-7-phosphate in GDP-D-glycero-α-D-manno-heptose biosynthetic pathway. The D-glycero-α-D-manno-heptose is a major constituent of lipopolysaccharide and contributes to virulence and antibiotic resistance to mycobacteria. In current study, we have performed the structural and biochemical analysis of *M. tuberculosis* GmhA, the first enzyme involved in D-sedoheptulose 7-phosphate isomerization in GDP-D-α-D-heptose biosynthetic pathway. The *Mtb*GmhA enzyme exits as tetramer and small angle X-ray scattering analysis also yielded tetrameric envelope in solution. The *Mtb*GmhA enzyme binds to D-sedoheptulose 7-phosphate with *K*_*m*_ ~ 0.31 ± 0.06 mM^−1^ and coverts it to D-glycero-D-α-manno-heptose-7-phosphate with catalytic efficiency (*k*_*cat*_*/K*_*m*_) ~ 1.45 mM^−1^ s^−1^. The residues involved in D-sedoheptulose 7-phosphate and Zn^2+^ binding were identified using modeled *Mtb*GmhA + D-sedoheptulose 7-phosphate + Zn^2+^ structure. To understand the role in catalysis, six site directed mutants of *Mtb*GmhA were generated, which showed significant decrease in catalytic activity. The circular dichroism analysis showed ~ 46% α-helix, ~ 19% β-sheet and ~ 35% random coil structures of *Mtb*GmhA enzyme and melting temperature ~ 53.5 °C. Small angle X-ray scattering analysis showed the tetrameric envelope, which fitted well with modeled *Mtb*GmhA tetramer in closed conformation. The *Mtb*GmhA dynamics involved in D-sedoheptulose 7-phosphate and Zn^2+^ binding was identified using dynamics simulation and showed enhanced stability in presence of these ligands. Our biochemical data and structural knowledge have provided insight into mechanism of action of *Mtb*GmhA enzyme, which can be targeted for novel antibiotics development against *M. tuberculosis.*

## Introduction

**Figure 1:**
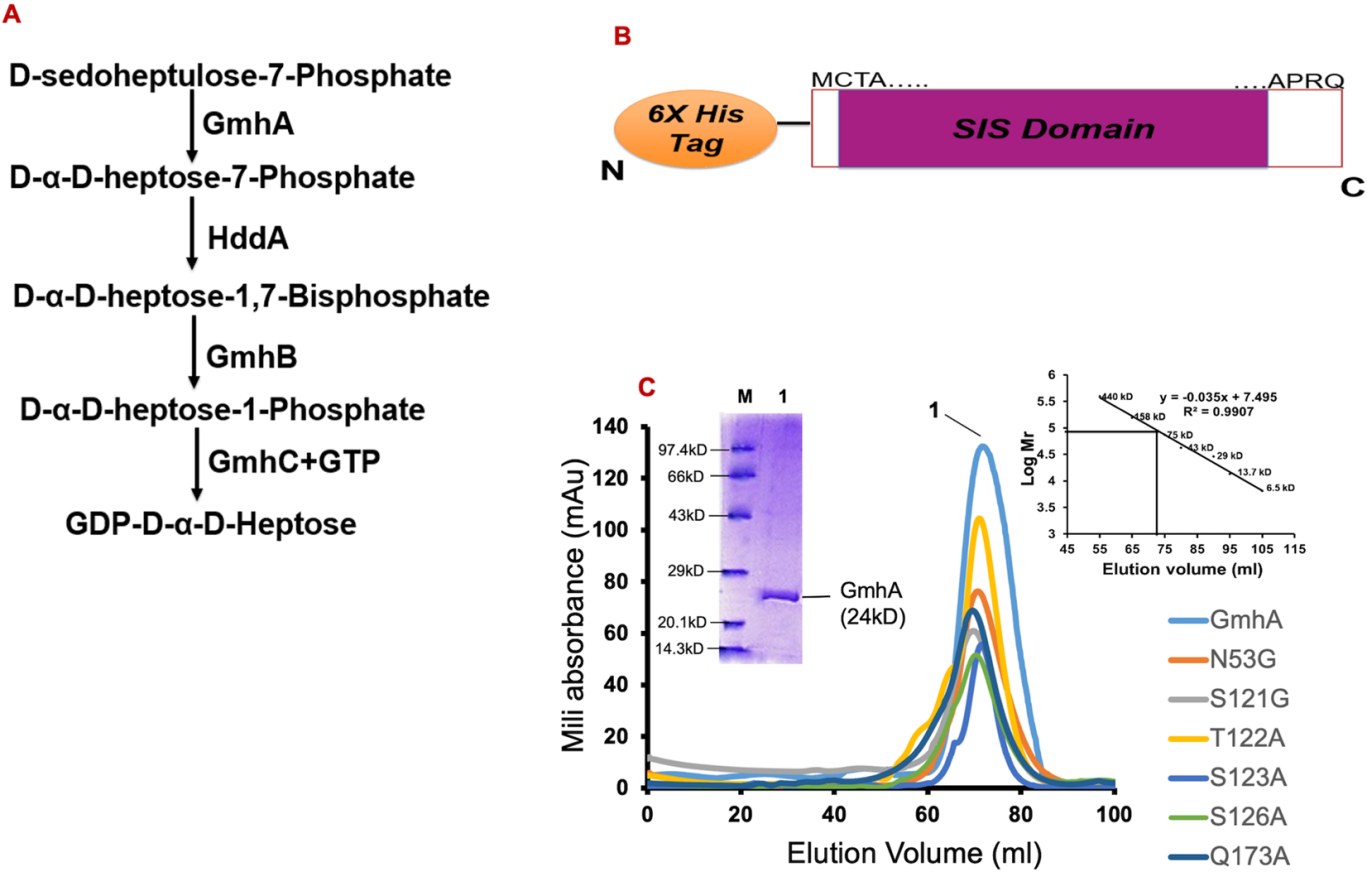
(**A**) Schematic diagram showing various enzymes involved in *M. tuberculosis* GDP-D-α-D-heptose biosynthetic pathway. (**B**) The gene construct used for *Mtb*GmhA enzyme expression. SIS domain represents the sugar isomerase domain. (**C**) The elution profile of wild type and six mutants of *Mtb*GmhA enzymes obtained from Superdex 200 column. Inset shows the SDS-PAGE of wild type enzyme; M = Mw marker, 1 = *Mtb*GmhA. The molecular mass of *Mtb*GmhA was calculated from standard curve generated using elution profiles of known molecular mass proteins.

The GDP-D-glycero-α-D-manno-heptose is a key building block of lipopolysaccharide in mycobacteria and blocking of its biosynthetic pathway leads to high antibiotic susceptibility and reduced virulence of mycobacteria. The enzymes involved in GDP-D-α-D-heptose biosynthetic pathway offer an attractive target for novel antibiotics development. The GDP-D-α-D-heptose in mycobacteria is synthesized in four steps, (1) isomerization of D-sedoheptulose 7-phosphate into D-glycero-D-α-manno-heptose-7-phosphate by GmhA enzyme (2) Phosphorylation of D-glycero-D-α-manno-heptose-7-phosphate at C1 position by HddA enzyme, which forms D-glycero-D-α-manno-heptose-1,7-bisphosphate (3) Removal of phosphate at C7 position in D-glycero-D-α-manno-heptose-1,7-bisphosphate by GmhB enzyme, which leads to D-glycero-D-α-manno-heptose-1-phosphate and (4) modification of the phosphate at C1 position to form a phosphodiester linkage with GDP by HddC enzyme and leads to GDP-D-glycero-α-D-manno-heptose (Fig. 1A). GDP-D-α-D-heptose is incorporated in the S-layer glycoproteins of mycobacterial membrane by specific precursor.

The heptose sugar maintains the structural integrity of the outer membrane of bacteria and interacts with membrane proteins and divalent cations. The heptose sugar is observed in inner core of lipopolysaccharide of *E. coli* and *P. aeruginosa*^1^. The heptose moiety is involved in ionic interactions and provides barrier to the passage of detergent, dyes and antibiotics through bacterial cell wall^2^ and is essential for survival of *P. aeruginosa*^3^.

The Gram-positive bacterial species contain crystalline two dimensional protein arrays, known as S- layers^4^. In S-layers, protein clusters are covalently attached to glycan chains, made up of 20–50 identical repeating units of α-L-rhamnose and D-glycero-α-D-manno-heptose units. Glycosylation of bacterial surface proteins with heptose and rhamnose are significant and involved in many functions e. g. adherence^5^, evasion of host immune response and enhanced resistance to proteolytic attack.

The GDP-D-α-D-heptose biosynthetic pathway in *M. tuberculosis* was first discovered by Eidels and Osborn^6^*.* The *M. tuberculosis* is a Gram-positive organism, however exhibits both features of Gram-positive and Gram-negative bacteria^7^. The GmhA is the key enzyme and highly conserved in Gram-positive and Gram-negative organisms e. g. *A*. *thermoaerophilus* and *C. acetobutylicum*. The *Mtb*GmhA showed 26.2% sequence identity with C-terminal region of L-glutamine-D-fructose-6-phosphateamido transferase, a ketose/aldose isomerase consists of sugar isomerase domain^8^. The D-glycero-α-D-manno-heptose is also observed as cellular component of *A. thermoaerophilus* DSM 10155, a member of the bacillus/clostridium group of Gram-positive organism^9^.

Crystal structures of GmhA enzymes from eight Gram-negative species have been determined^10–13^. The GmhA enzymes from *P. aeruginosa* and *V. cholerae* were crystallized as dimer, while other GmhA orthologues were crystallized as tetramer in the asymmetric unit. The GmhA tetramer is found in two distinct conformations, “closed” and “open”. The “open” conformation is characterized by an extended α3–β2 loop, less ordered α3′ region, and loosely packed dimer-dimer interface. In “closed” conformation, unstructured α3′ region adopts a helical structure and α3–β2 loop is positioned inward, which allowed more extensive dimer-dimer interaction.

In current study, we have performed biochemical and structural analysis of *Mtb*GmhA enzyme. The *Mtb*GmhA enzyme was purified and its activity was analyzed by coupled assay involving *Mtb*GmhA, *Mtb*HddA and *Mtb*GmhB enzymes. The binding analysis between all three enzymes was performed using surface plasmon resonance technique. The *Mtb*GmhA residues involved in D-sedoheptulose 7-phosphate and Zn^2+^ binding were identified using modeled complex and their roles in catalysis were determined using site directed mutagenesis. The circular dichroism, small angle X-ray scattering, and dynamics simulation techniques were used to analyze the *Mtb*GmhA structure and dynamics involved in D-sedoheptulose 7-phosphate and Zn^2+^ binding. Our structural and biochemical analysis on *Mtb*GmhA explained the mechanism of action, which will contribute in the development of novel antibiotics against *M. tuberculosis*.

## Results and discussion

### Purified *Mtb*GmhA forms tetramer in solution

The Fig. 1A showed the various steps involved in GDP-D-α–D-heptose biosynthetic pathway in *M. tuberculosis.* The *Mtb*GmhA enzyme (196 residues, Mw ~ 24 kDa) is the first enzyme involved in D-sedoheptulose 7-phosphate isomerization and converts it to D-glycero-D-α-manno-heptose-7-phosphate. The *Mtb*GmhA gene was cloned in *pET28a* expression vector (Fig. 1B) and overexpressed in soluble fraction of *Escherichia coli.* The *Mtb*GmhA enzyme was purified and eluted as tetramer (~ 100 kDa) from Superdex 200 (16/60) column, identified based on molecular mass standard (Fig. 1C, inset). The purified *Mtb*GmhA showed more than 98% purity on SDS-PAGE (Fig. 1C, inset). The SDS-PAGE profile of 9 eluted fractions of *Mtb*GmhA (E1–E9) are shown in Fig. S1. We have generated the six-point mutants e. g. N53G, S121G, T122G, S123G, S126G, Q173G of *Mtb*GmhA enzyme and all mutant proteins were purified like wild type enzyme and eluted as tetramer (Fig. 1C). The primers used for gene amplification of six *Mtb*GmhA mutants are shown in Table S1. PDBePISA server analysis also indicated that biological assembly of *Mtb*GmhA enzyme is a tetramer^12^.

The GmhA enzymes from *P. aeruginosa* and *V. cholera* form tetramer in solution^10,14^. However, their crystallographic asymmetric unit contains dimer and functional tetramers were obtained using two-fold crystallographic symmetry. The *H. pylori* GmhA is the only homologue, which was observed as homodimer in solution^15,16^.

### Small angle X-ray scattering analysis revealed the tetrameric envelope of *Mtb*GmhA enzyme

**Figure 2:**
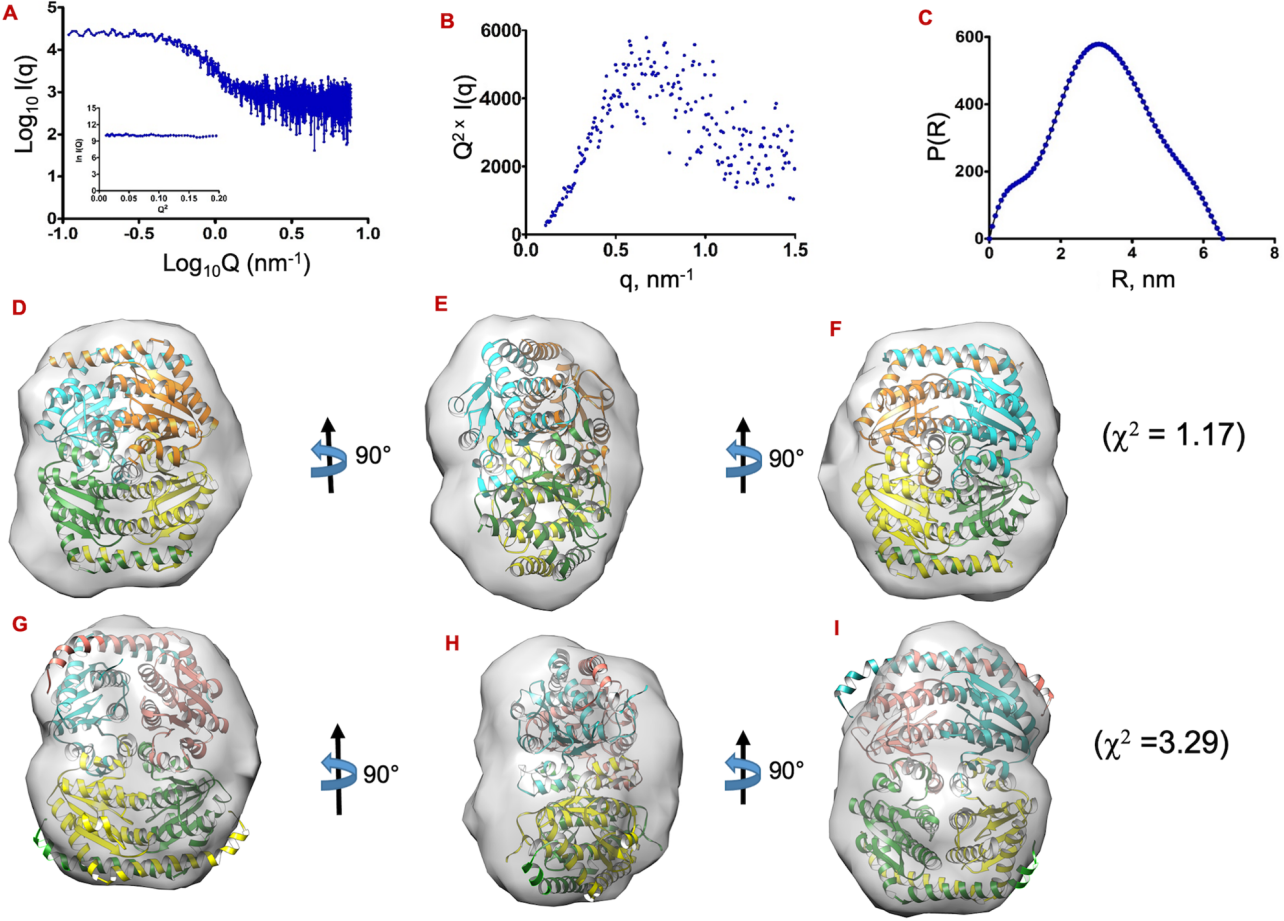
Small angle X-ray scattering analysis on *Mtb*GmhA enzyme. (**A**) SAXS intensity profile of *Mtb*GmhA. The inset shows linear fit to Guinier region of measured data. (**B**) Kratky plot of *Mtb*GmhA. (**C**) The P(R) curves computed for *Mtb*GmhA indicating the frequency distribution of interatomic vectors in predominant scattering species. (**D**–**F**) Fitting of *Mtb*GmhA tetramer (closed state) in shape restored from dummy atom modeling using small angle X-ray scattering data and shown after every 90° rotation along Y-axis. Fitting of *Mtb*GmhA tetramer (open state) in shape restored from dummy atom modeling using small angle X-ray scattering data and shown after every 90° rotation.

The small angle X-ray scattering data was collected on *Mtb*GmhA enzyme to analyze its low-solution shape in solution. The SAXS intensity profile [I(Q)] as a function of momentum transfer vector Q, Guinier plot using globular approximation, Kratky plot and pair-distribution function [P(R)] were calculated (Fig. 2A–C). The Fig. 2A showed the double logarithmic plot of small angle X-ray scattering data (log_10_ I(Q) ~ log_10_Q). These data showed no inter particulate effect or aggregation of protein, as lack of upwards or downwards points were observed, when data approached to 0 nm^−1^. The Fig. 2B showed the bell-shaped peak in Kratky plot, which indicated the globular shape of *Mtb*GmhA in solution. The Guinier plot analysis yielded the radius of gyration R_G_ ~ 2.38 ± 0.11 nm, quite similar to theoretical R_G_ of *Mtb*GmhA tetramer. The Fig. 2C showed the probability distribution of various interatomic vectors P(R), using small angle X-ray scattering data as a reference. These data yielded the maximum linear dimension (D_max_) ~ 6.35 nm and R_G_ ~ 2.45 nm for *Mtb*GmhA tetramer (Fig. 2C). The single peak in P(R) profile indicated the globular shape of *Mtb*GmhA enzyme. Using Lysozyme data (conc. ~ 1 mg/ml), the intensity at zero angle (I_0_) ~ 464 a. u. was observed for 1.0 kDa in 1 h exposure. Using it as control, the relative intensity of *Mtb*GmhA protein (conc. ~ 1.2 mg/ml) has yielded the molecular mass ~ 93.2 kDa, which corresponds to *Mtb*GmhA tetramer **(**Table 1).

**Table 1:** Small angle X-ray scattering data collection and experimentally derived parameters of *Mtb*GmhA. R_g_ = radius of gyration. D_max_ = maximum particle dimension.

	*Mtb*GmhA
**Data collection parameters**	
Instrument	SAX Space (Anton Paar)
Beam geometry	10 mm slit
Wavelength (Å)	1.5418
Q range (nm^−1^)	0.10–3.00
Exposure time (min)	30
Concentration (mg/ml)	1.22
Temperature (K)	283
**Structural parameters**	
I(0) (cm^−1^) [from P(R)]	25,650.00
R_g_ (nm) [from P(R)]	2.73 ± 0.11
I(0) (cm^−1^ from Guinier)	26,737.10 ± 492.71
R_g_ (nm) (from Guinier)	2.48 ± 0.17
D_max_ (nm)	7.9
Porod volume estimate (nm^3^)	130.29
**Molecular-mass determination**	
Molecular mass M_r_ (kDa) I(0)	93.2
Calculated monomeric M_r_ from sequence (KDa)	24.105
**Software employed**	
Primary data reduction	SAXSquant
Data processing	PRIMUSQT
Ab initio analysis	DAMMIF
Validation and averaging	DAMAVER
Computation of model intensities	CRYOSOL
3D graphics representations	PyMOL

Uniform density modeling protocol was used to restore the shape, which yielded 10 independents *Mtb*GmhA models. All models were averaged, refined and yielded the normalized spatial disposition (NSD) ~ 1.30 ± 0.03, a measure of similarity between individual solutions. The NSD showed the high similarity between dummy residue shape of solved models. The modeled *Mtb*GmhA tetramer in closed state fitted well into SAXS envelope (χ^2^ ~ 1.17) (Fig. 2D–F), compared to open state (χ^2^ ~ 3.29) (Fig. 2G–I). The CRYSOL program was used to compute the theoretical small angle X-ray scattering profile from *Mtb*GmhA tetrameric model and compared with experimental small angle X-ray scattering data. These data indicated that *Mtb*GmhA exists as monodisperse, homogeneous and closed state tetramer in solution.

### Activity assay using wild type and mutant *Mtb*GmhA enzymes showed the roles of various active site residues

#### Activity assay using wild type enzyme

**Figure 3:**
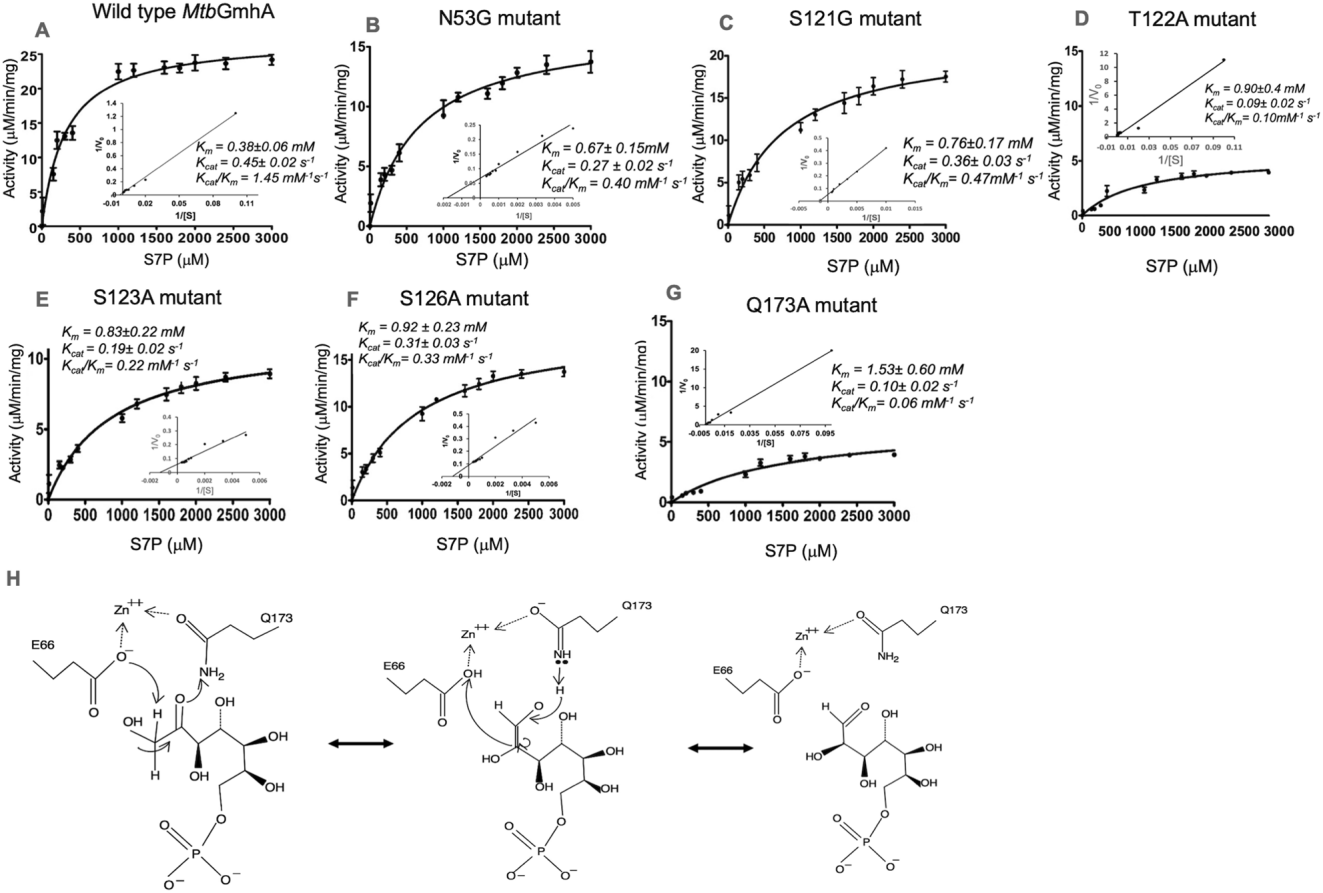
Activity assay on wild type and six mutants of *Mtb*GmhA enzymes. Three replicates were performed for each measurement. The *Mtb*GmhA activity was determined using Malachite green phosphate detection kit. A coupling reaction was performed using *Mtb*GmhA, *Mtb*HddA and *Mtb*GmhB enzymes and release of inorganic phosphate was monitored at 600 nm. Following saturation curves were obtained, (**A**) wild type *Mtb*GmhA (**B**) N53G mutant (**C**) S121G mutant (**D**) T122A mutant (**E**) S123A mutant (**F**) S126A mutant and (**G**) Q173A mutant. The 0 to 3 mM concentrations of D-sedoheptulose 7-phosphate were used in all assays and results are given in Table 2. (**H**) Proposed mechanism of *Mtb*GmhA used in isomerization of D-sedoheptulose 7-phosphate to D-glycero-D-α-manno-heptose-7-phosphate through enediol intermediate, in which Gln173 acted as a catalytic acid and forms an imine stabilized by Zn^2+^ ion and Glu65 acted as catalytic base.

For activity analysis, the *Mtb*HddA and *Mtb*GmhB enzymes were purified (data not shown), performed the coupling assay using all three enzymes and monitored the release of phosphate. As control, a phosphate standard curve was calculated using 0–50 μM phosphate, dissolved in coupling reaction buffer having no *Mtb*GmhA enzyme. Following kinetic parameters, *K*_*m*_ ~ 0.31 ± 0.06 mM^−1^, *k*_*cat*_ ~ 0.45 ± 0.02 s^−1^ and catalytic efficiency (*k*_*cat*_*/K*_*m*_) ~ 1.45 mM^−1^ s^1^ were obtained for *Mtb*GmhA enzyme (Fig. 3A, Table 2).

**Table 2:** Kinetic parameters of wild type and six *Mtb*GmhA mutants obtained using Michaelis Menten curve and Lineweaver–Burk plot (in red bracket). The kinetic parameters of *E. coli.* GmhA, *B. pseudomallei* GmhA and *H. pyroli* GmhA are shown for comparative analysis.

Protein	*K*_*m*_ (mM)	*k*_*cat*_ (s^−1^)	*k*_*cat*_*/K*_*m*_ (mM^−1^ s^−1)^
***MtbGmhA***	0.31 ± 0.06 (0.38)	0.45 ± 0.02 (0.52)	1.45 (1.36)
N53G mutant	0.67 ± 0.15 (0.62)	0.27 ± 0.02 (0.27)	0.40 (0.43)
S121G mutant	0.76 ± 0.17 (0.73)	0.36 ± 0.03 (0.33)	0.47 (0.45)
S123A mutant	0.92 ± 0.23 (0.93)	0.31 ± 0.03 (0.33)	0.33 (0.35)
S126A mutant	0.83 ± 0.22 (0.83)	0.19 ± 0.02 (0.18)	0.22 (0.21)
T122A mutant	0.90 ± 0.4 (0.89)	0.09 ± 0.02 (0.11)	0.10 (0.12)
Q173A mutant	1.53 ± 0.6 (1.54)	0.10 ± 0.02 (0.13)	0.06 (0.08)
***E. coli GmhA***	0.9 ± 0.3	0.4 ± 0.7	0.5
***B. pseudomallei GmhA***	0.4 ± 0.2	0.5 ± 0.1	1.2
***H. pylori GmhA***	0.5 ± 0.1	33.1 ± 2.1	71.9

The *Mtb*GmhA kinetic parameters were compared with other GmhA orthologues, as shown in Table 2. The catalytic efficiency of *Mtb*GmhA enzyme was quite similar to *B. pseudomallei* and *E. coli* GmhA enzymes. However, *H. pylori* GmhA enzyme showed ~ 50-fold higher catalytic efficiency (*k*_*cat*_*/K*_*m*_) than *Mtb*GmhA enzyme and other GmhA orthologues. The *H. pylori* GmhA was observed as dimer in solution, while tetramers were observed for GmhA enzymes from *M. tuberculosis*, *E. coli* and *B. pseudomallei*. It is not clear how oligomeric state of GmhA enzyme affects its catalytic efficiency.

#### Activity assay using six *Mtb*GmhA mutants

We build the *Mtb*GmhA + S7P + Zn^2+^ complex and identified the six residues involved in D-sedoheptulose 7-phosphate binding. we have generated the six *Mtb*GmhA mutants, performed coupling assay and release of free P_i_ was monitored (Fig. 3B–G, Table 2).

As seen in Table 2, Gln173 → Ala mutation leads to ~ 24 fold decrease in catalytic efficiency and ~ fivefold decrease in binding affinity to D-sedoheptulose 7-phosphate substrate in *Mtb*GmhA, when compared to wild enzyme. Following data e. g. Asn53 → Gly mutation (~ twofold decrease in binding affinity and ~ 3.5 fold decrease in catalytic efficiency), Ser121 → Gly mutation (~ twofold decrease in binding affinity and ~ 3.5 fold decrease in catalytic efficiency), Thr122 → Ala mutation (~ threefold decrease in binding affinity and ~ 14.5 fold decrease in catalytic efficiency), Ser123 → Ala mutation (~ threefold decrease in binding affinity and ~ 4.5 fold decrease in catalytic efficiency), Ser126 → Ala mutation (~ threefold decrease in binding affinity and ~ 4.3 fold decrease in catalytic efficiency) were observed. The Asn53 forms bifurcated hydrogen bonds with O_4_ and O_9_ atoms of D-sedoheptulose 7-phosphate and Asn53 → Gly mutation leads to ~ 3.5 fold decrease in catalytic efficiency. The Gln173 forms hydrogen bond with O_2_ atom of D-sedoheptulose 7-phosphate and its mutation to Ala leads to ~ 24 fold decrease in catalytic efficiency. The bar diagram showing the catalytic efficiency of wild type and six *Mtb*GmhA mutants are shown in Fig. S2.

In *Escherichia coli and P. aeruginosa* GmhA structures^10^*,* the Glu68 and His183 acted as acid and base in enzyme catalysis, which converted the D-sedoheptulose 7-phosphate to D-glycero-D-α-manno-heptose-7-phosphate. In *B. pseudomallei* GmhA structure^12^, His183 was buried behind Zn^2+^ in the active site and Glu68 and Gln175 bind to Zn^2+^ in such a way, that their side chains acted as acid and base in enzyme catalysis.

In modeled *Mtb*GmhA + D-sedoheptulose 7-phosphate + Zn^2+^ tetramer, the Glu66 from one monomer and Gln173 from another monomer bind to D-sedoheptulose 7-phosphate and Zn^2+^ and may act as acid and base in enzyme catalysis (Fig. 3H). Mutation of Gln173 → Ala has shown ~ 50 fold decrease in catalytic efficiency of *Mtb*GmhA enzyme, indicating its involvement in enzyme catalysis. Similar mechanism has been observed in other GmhA orthologues, which contain Zn^2+^ in the active site.

### *MtbGmhA, MtbHddA and MtbGmhB* enzymes interact each other in µM range

**Figure 4:**
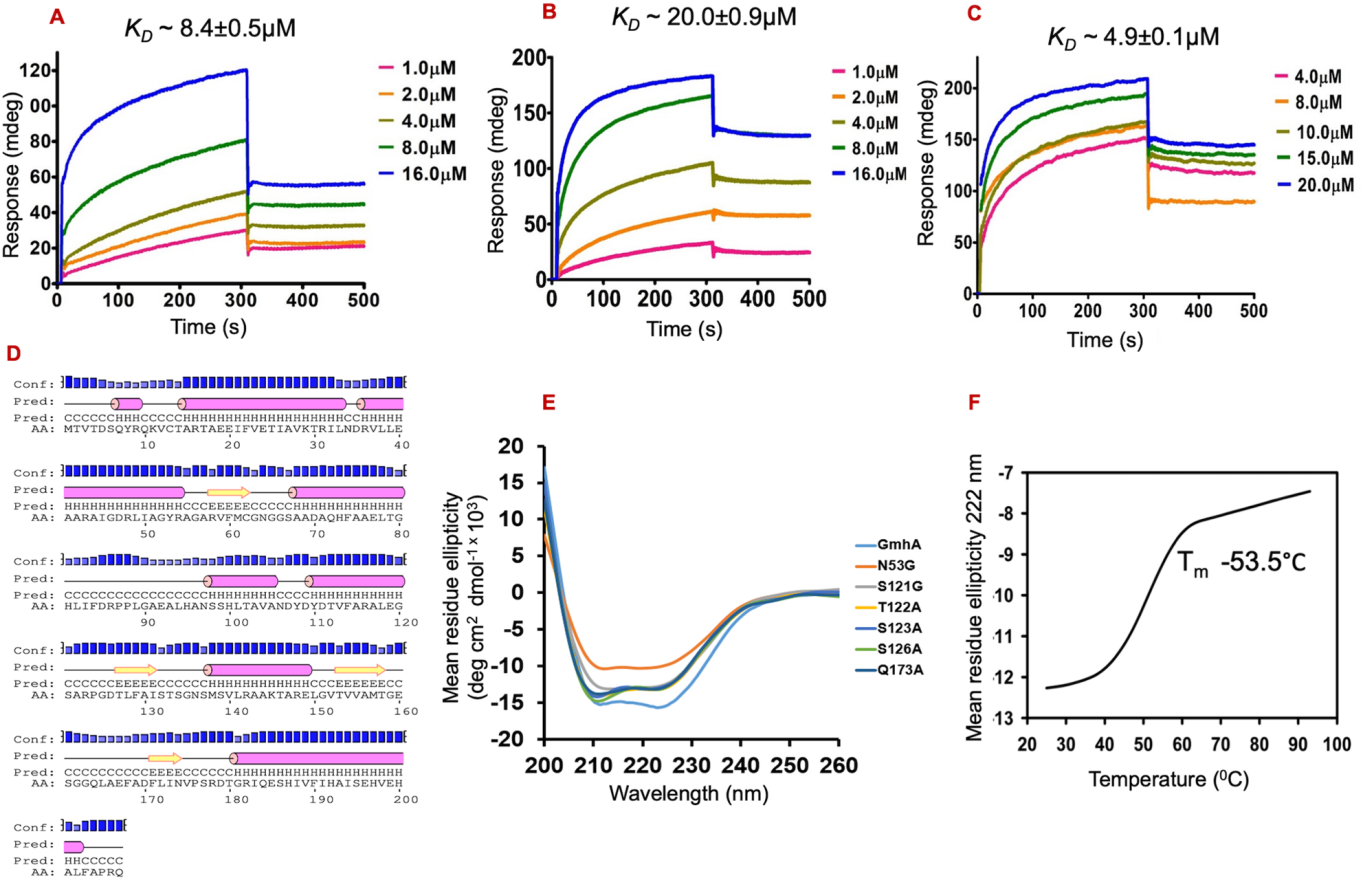
Interaction analysis between *Mtb*GmhA, *Mtb*GmhB and *Mtb*HddA enzymes using surface plasmon resonance technique. (**A**) Sensogram showing the binding of *Mtb*HddA on immobilized *Mtb*GmhA. Five different concentrations of *Mtb*HddA enzyme (1–16 µM) were used in this assay, which yielded the *K*_*D*_ = 8.45 ± 0.45 µM. (**B**) Sensogram showing binding of *Mtb*HddA on immobilized *Mtb*GmhB. Five different concentrations of *Mtb*GmhB (1–16 µM) were used in this assay, which yielded the *K*_*D*_ = 20 ± 0.95 µM. (**C**) Sensogram showing the binding of *Mtb*GmhB on immobilized *Mtb*GmhA. Five different concentrations of *Mtb*GmhB (4 to 20 µM) were used in current assay, which yielded *K*_*D*_ = 4.9 ± 0.11 µM. (**D**) Psi-Pred program analysis on *Mtb*GmhA sequence showing the secondary structural contents in *Mtb*GmhA enzyme. (**F**) The circular dichroism data of wild type and six *Mtb*GmhA mutants, shown in different colors. (**G**) Thermal denaturation profile on *Mtb*GmhA enzyme, indicating the melting temperature ~ 53.5 °C of the enzyme.

Surface plasmon resonance technique was used to analyze the binding affinity between *Mtb*GmhA, *Mtb*HddA and *Mtb*GmhB enzymes. Following *K*_*D*_ values were observed e. g. ~ 8.4 ± 0.5 µM between *Mtb*GmhA and *Mtb*HddA (Fig. 4A), 20.0 ± 0.9 µM between *Mtb*HddA and *Mtb*GmhB (Fig. 4B) and 4.9 ± 0.1 µM between *Mtb*GmhA and *Mtb*GmhB (Fig. 4C). These data indicated that all three enzymes bind each other in µM range and form a stable complex in GDP-D-α-D-heptose biosynthetic pathway.

The genetic organization in D-α-D-heptose biosynthetic pathway was first described in *A. thermoaerophilus* DSM 10155, in which all enzymes were located as cluster containing genes involved in synthesis and transfer of dTDP-rhamnose^9^. In *M. tuberculosis*, the genes for two different pathways were present at different locations and involved in synthesis of glycolipids and protein glycosylation. Interestingly, in CDC 1551 strain of *M. tuberculosis*, the GmhA and GmhB genes were fused as single gene, probably encoding a bifunctional enzyme having isomerase and phosphatase activities.

### Circular dichroism analysis revealed the secondary structures and thermal stability of *Mtb*GmhA enzyme

The PSIPRED program analysis on *Mtb*GmhA sequence showed the secondary structures of enzyme (Fig. 4D). The Far-UV CD data on *Mtb*GmhA and its six mutants were collected in 260–200 nm range (Fig. 4E). The K2D program has yielded the ~ 46% α-helix, ~ 19% β-sheet and ~ 35% random coil structures of wild type *Mtb*GmhA, quite similar to structures observed in six *Mtb*GmhA mutants (Fig. 4E, Table 3). Various secondary structure prediction programs have yielded quite similar secondary structures in *Mtb*GmhA enzyme, as observed in CD analysis (Table S2).

**Table 3:** Secondary structural contents in six mutants of *Mtb*GmhA obtained using circular dichroism spectroscopy.

Protein	α-helix (%)	β-sheet (%)	Random coil (%)
N53G mutant	46	21	33
S121G mutant	47	17	37
T122A mutant	38	16	46
S123A mutant	39	15	46
S126A mutant	40	17	43
Q173A mutant	39	16	45

For thermal stability analysis, the mean residue ellipticity (θ_222_) data was collected on *Mtb*GmhA enzyme in 25–90 °C range with 10 °C step (Fig. 4F). The helical structure of *Mtb*GmhA enzyme was quite stable till 42 °C and disordered at 65 °C. A melting temperature, T_m_ ~ 53.5 °C was obtained, which indicated the high thermostability of *Mtb*GmhA enzyme.

### The modeled ***Mtb***GmhA + D-sedoheptulose 7-phosphate + Zn^2+^ tetramer showed the active site involved in D-sedoheptulose 7-phosphate and Zn^2+^ binding

**Figure 5:**
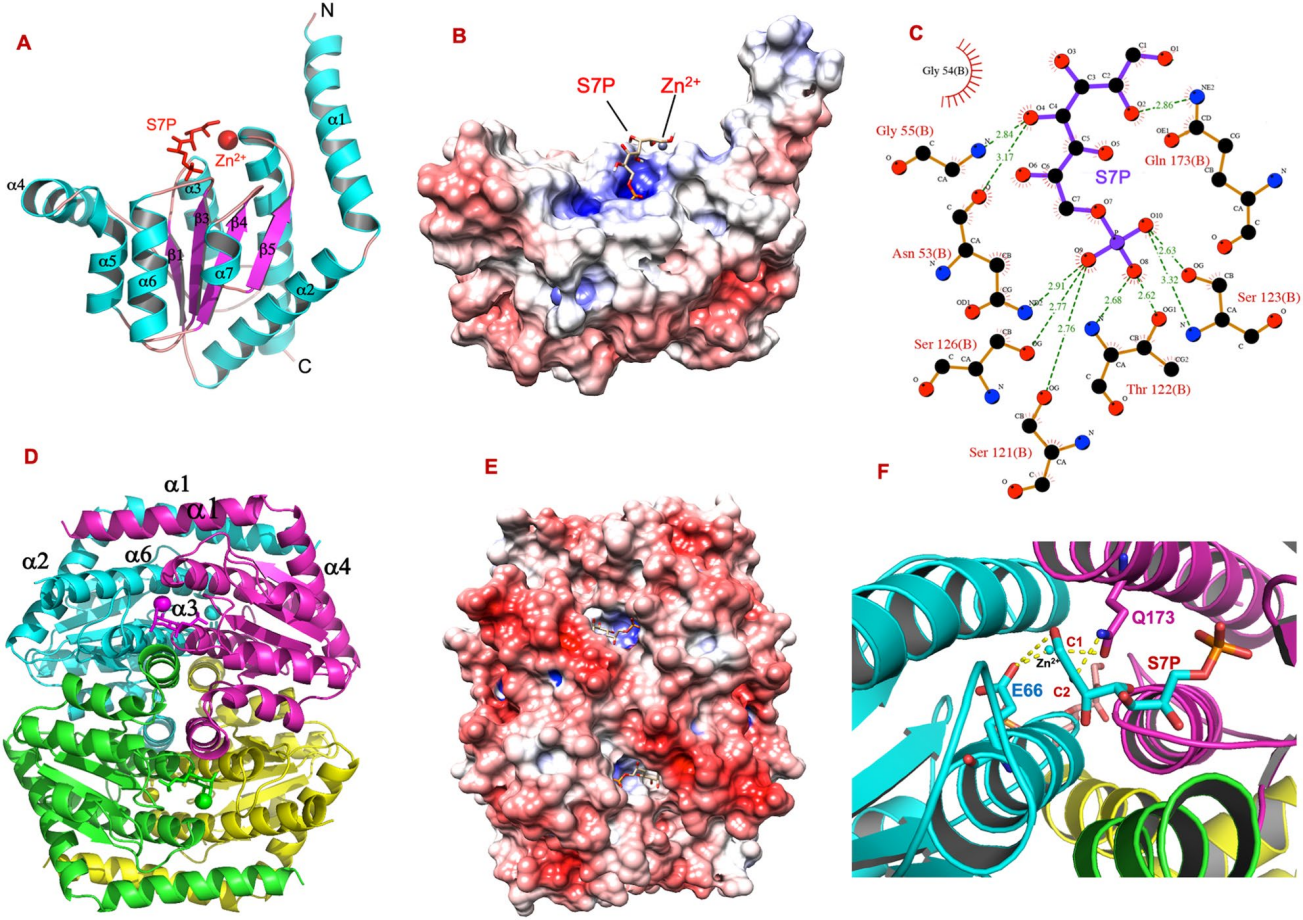
(**A**) The *Mtb*GmhA + D-sedoheptulose 7-phosphate (S7P) + Zn^2+^ complex model showing the α-helices (in cyan), β-sheets (in magenta), loops (in orange). The D-sedoheptulose 7-phosphate and Zn^2+^ ion are shown in red colors. (**B**) Electrostatic surface diagram of *Mtb*GmhA monomer showing the D-sedoheptulose 7-phosphate and Zn^2+^ ion in the active site (blue positive surface). (**C**) LigPlot analysis of *Mtb*GmhA + D-sedoheptulose 7-phosphate + Zn^2+^ complex showing the hydrogen bonds and van der Waals interactions between D-sedoheptulose 7-phosphate and Zn^2+^ with active site residues of *Mtb*GmhA enzyme. The green dashed lines showed the hydrogen bonds and residues in “arc with spikes” are involved in van der waals interactions with ligands. (**D**) *Mtb*GmhA + D-sedoheptulose 7-phosphate + Zn^2+^ tetramer showing each monomer in different color and D-sedoheptulose 7-phosphate and Zn^2+^ ligands in the active site of each monomer. (**E**) Electrostatic surface diagram of *Mtb*GmhA tetramer showing four catalytic clefts (blue, positive charge), which accommodate the D-sedoheptulose 7-phosphate and Zn^2+^ in their binding pockets. (**F**) Interface between two *Mtb*GmhA monomers, in which Glu66 residue (cyan) from one monomer and Zn^2+^ and Gln173 residue from another monomer binds to O1-C1 and O2-C2 of D-sedoheptulose 7-phosphate respectively and proposed as involved in isomerization of D-sedoheptulose 7-phosphate to D-glycero-D-α-manno-heptose-7-phosphate.

The *Mtb*GmhA model (1–196 residues) was obtained using I-TASSER (Iterative Threading ASSEmbly Refinement) server, which used the PDB-2X3Y (Crystal structure of *B. pseudomallei* GmhA tetramer in closed state^12^) as the best input template (RMSD = 0.79, id1 = 0.39, id2 = 0.39, Conv = 0.98, Z-score = 3.2). The I-TASSER server also used the PDB-2I2W (Crystal structure of *Escherichia Coli* phosphoheptose isomerase in open state^10^) as another template for *Mtb*GmhA modeling. However, current template did not yield suitable *Mtb*GmhA model, as having following parameters (RMSD = 2.1, id1 = 0.35, TM-score = 0.82, Conv = 0.89). The D-sedoheptulose 7-phosphate and Zn^2+^ ions were docked into *Mtb*GmhA tetramer using GLIDE module of Schroedinger program (Fig. 5A). A docking score of -5.44 and X-score of 8.1 were observed in docking analysis. The *P. seudomallei* GmhA structures (PDB-2X3Y and PDB-2XBL) were used as reference to validate the results obtained in docking analysis of both ligands.

The *Mtb*GmhA monomer consists of central five stranded parallel β-sheets, flanked by eight α-helices and forms a helix-beta-helix sandwich (Fig. 5A). Overall fold of *Mtb*GmhA enzyme was quite similar to flavodoxin-type nucleotide-binding motif, as observed in other GmhA orthologues. The Electrostatic surface of *Mtb*GmhA monomer (Fig. 5B) showed the positively charged surface at catalytic cleft, which accommodates the D-sedoheptulose 7-phosphate and Zn^2+^ ion. The LIGPLOT v.4.5.3 analysis of *Mtb*GmhA + D-sedoheptulose 7-phosphate + Zn^2+^ complex (Fig. 5C) showed that D-sedoheptulose 7-phosphate forms hydrogen bonds with Asn53 and Gly55 residues of β1–α3 helix region, Ser121, Thr122, Ser123 and Ser126 residues from β3–α6 helix region, Gln173 residue from α8 helix in *Mtb*GmhA enzyme. The Zn^2+^ ion forms hydrogen bond with Gln173 of *Mtb*GmhA, which stabilize the D-sedoheptulose 7-phosphate in the active site. The *C. jejuni* and *P. aeruginosa* GmhA enzymes also adopt the closed structures^14^ and metal binding sites were observed in both enzymes. It appears that Zn^2+^ binding induces the closed state conformation of GmhA and more suitable for D-sedoheptulose 7-phosphate binding and catalysis.

Since *Mtb*GmhA is observed as tetramer in solution, we build the *Mtb*GmhA + D-sedoheptulose 7-phosphate + Zn^2+^ tetramer using the same PDB-2X3Y as input template (Fig. 5D). The *Mtb*GmhA tetramer forms a compact structure, in which four α-helices form hexagons, tilted at 120° and may help in structural stabilization. The α4 and α5 helices of each *Mtb*GmhA monomer facing each other and forms a compact groove. 50% of α1 helix of one monomer interacts with α1 helix of the neighboring monomer. The Electrostatic surface of the *Mtb*GmhA tetramer (Fig. 5E) showed the positively charged surface at catalytic pockets involved in D-sedoheptulose 7-phosphate and Zn^2+^ ion binding. Overall surface of *Mtb*GmhA tetramer was negatively charged, except D-sedoheptulose 7-phosphate and Zn^2+^ binding pockets. The loop regions connecting β1–α3 and β3–α6 strands were involved in formation of D-sedoheptulose 7-phosphate binding pocket in *Mtb*GmhA enzyme. The Fig. 5F showed the Glu66 residue of one monomer (cyan) bind to O1-C1 of D-sedoheptulose 7-phosphate and Gln173 residue of another monomer (Pink) involved in binding to O2–C2 of D-sedoheptulose 7-phosphate and Zn^2+^ and involved in enzyme catalysis.

### Differences between *Mtb*GmhA and other known GmhA enzymes

#### Sequence alignment and active site analysis

**Figure 6:**
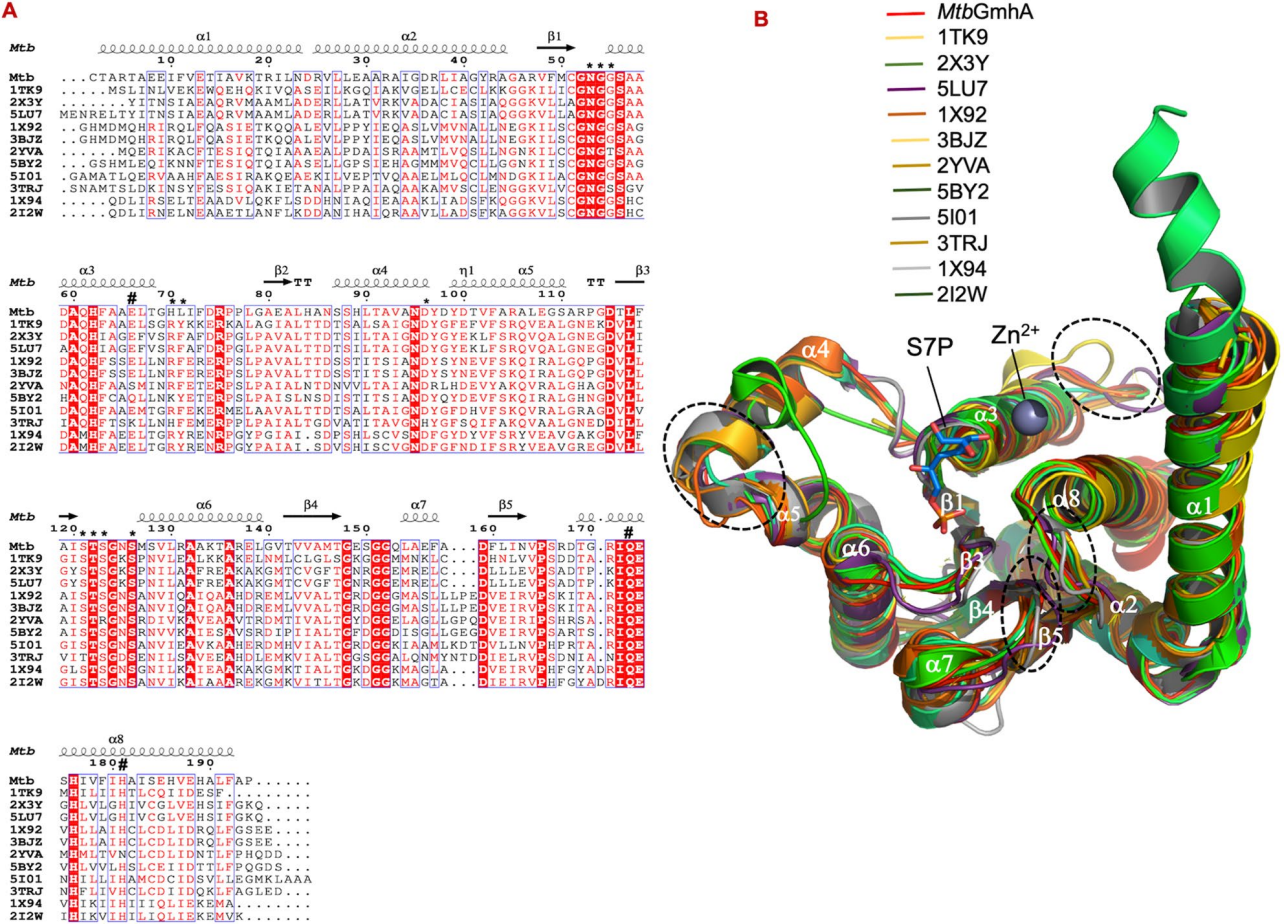
(**A**) Multiple sequence alignment of *Mtb*GmhA with eleven GmhA orthologues, which showed highly conserved (red shade), semi-conserved (red letter) and dispersive residues (black letter). The residues involved in D-sedoheptulose 7-phosphate binding (*), in Zn^2+^ binding and enzyme catalysis (#) are shown above the sequence alignment. (**B**) Structural superposition of 11 GmhA orthologues on *Mtb*GmhA model. These structures are 1TK9, 2X3Y, 5LU7, 1X92, 3BJZ, 5BY2, 5IOL, 3TRJ, 1X94, 1I2W. The 2YVA is not a *Mtb*GmhA orthologue, but a homologue with different function. Major conformational changes are highlighted with dashed circles.

The Fig. 6A showed the sequence alignment of *Mtb*GmhA enzyme with 11 GmhA orthologues. The secondary structures of *Mtb*GmhA model were placed on the top of sequence alignment. The Asn53, Gly55, Ser121, Thr122, Ser123, Ser126 residues (*) involved in D-sedoheptulose 7-phosphate and Zn^2+^ binding were found quite conserved. The active site residues e. g. Gln173 from one monomer and Glu66 from another monomer (shown as #), which bind to C1-O1 and C2-O2 of D-sedoheptulose 7-phosphate were also quite conserved. Other *Mtb*GmhA residues involved i in substrate binding e. g. Asn53 (fully conserved), Gly55 (G → T in 2YVA, G → S in 3TRJ), Ser121 (S → T in 3TRJ), Thr122 (fully conserved), Ser123 (S → R in 2YVA), Ser126 (fully conserved ), Gln173 (fully conserved) and Glu66 (E → S in 2YVA, E → Q in 5BY2 and E → K in 3TRJ) have shown degree of conservation with 11 other GmhA orthologues. In *Francisella tularensis*, Ser121 was replaced with Thr121, however not involved in catalytic cleft formation^15,16^. Similarly, Ser123 → Arg mutation in DiaA helps in timely initiation of chromosomal replication during cell cycle. In DiaA structure, important residues involved in enzyme catalysis were Ser52, Ala53, Arg71, Pro72, Asn83, Lys101, Leu190, and Phe191^17^.

In addition, other *Mtb*GmhA residues (Gly52, Gly54, Ala60, His62, Arg75, Asn95, Asp115, T116 (T → V in 11 enzymes), L117, G124, A132, A136, G148, Gly151, Gly152, Asp159, Pro165, Ile172, Glu174, His176) were fully conserved in sequence alignment with 11 GmhA orthologues.

#### Structural superposition

Structural superposition of 11 GmhA orthologues on *Mtb*GmhA model have yielded following r.m.s. deviation e. g*.* 1TK9 (Crystal structures of two phosphate isomerases from *C. jejuni*)^14^ (0.65 Å, 146 Cα atoms), 2X3Y (Crystal structure of GmhA from *B. pseudomallei*)^12^ (0.27 Å, 168 Cα atoms), 5LU7 (Crystal structure of heptose isomerase GmhA mutants)^18^ (0.15 Å, 168 Cα atoms), 1X92 (Crystal structure of *Pseudomonas Aeruginosa* phosphoheptose isomerase in complex with reaction product D-glycero-D-mannopyranose-7-Phosphate)^10^ (0.56 Å, 148 Cα atoms), 3BJZ^10^ (0.75 Å, 133 Cα atoms), 2YVA^17^ (Crystal structure of *E. coli* DiaA) (0.76 Å, 147 Cα atoms), 5BY2^11^ (Sedoheptulose 7-phosphate isomerase from *Colwellia psychrerythraea* strain 34H) (0.63 Å, 147 Cα atoms), 5IOL (Crystal structure of Nucleoside Diphosphate kinase from *Schistosoma mansoni*)^19^ (0.53 Å, 147 Cα atoms), 3TRJ (Crystal structure of phosphoheptose isomerase from *Francisella tularensis*)^15,16^, 1X94 (Crystal structure of two putative phosphoheptose isomerase from *Vibrio cholerae*)^14^ (0.64 Å, 116 Cα atoms), 1I2W (Crystal structure of *Bacillus licheniformis* BS3 class-A beta-lactamase and acyl-enzyme adduct formed with cefoxtin)^20^ (0.69 Å, 128 Cα atoms) (Fig. 6B). Major changes were observed in the loop regions connecting α3–β2, α8–β5, β5–α6 strands and minor changes in other loops of *Mtb*GmhA enzyme. The β1–α3 loop is involved in D-sedoheptulose 7-phosphate and Zn^2+^ binding and found quite conserved in 11 GmhA orthologues and showed least conformation change. Major conformational changes were observed in α4 and α7 helices of *E. coli* GmhA enzyme (2I2W, open state) to *Mtb*GmhA, when compared to structures of 10 GmhA orthologues.

### Dynamic simulation on *Mtb*GmhA tetramer as (1) Apo (2) D-sedoheptulose 7-phosphate bound and (3) D-sedoheptulose 7-phosphate + Zn^2+^ bound state has revealed the dynamics involved in ligand recognition.

1 ns dynamics simulation was performed on *Mtb*GmhA tetramer in (i) Apo (ii) D-sedoheptulose 7-phosphate bound and (iii) D-sedoheptulose 7-phosphate + Zn^2+^ bound state and analyzed the dynamics involved in D-sedoheptulose 7-phosphate and Zn^2+^ binding (Table 4). All three *Mtb*GmhA tetramers obtained after dynamics simulation showed good stereochemistry and lie in allowed regions of Ramachandran plot (Figs. S3–S5).

**Table 4:** Dynamics simulation parameters of Apo, D-sedoheptulose 7-phosphate (D-S7P) bound and D-sedoheptulose 7-phosphate + Zn^2+^ (D-S7P + Zn^2+^) bound *Mtb*GmhA tetramer.

Models	Apo	D-S7P	D-S7P + Zn^2+^
	(Tetramer)	(Tetramer)	(Tetramer)
Protein atoms	11,444	11,444	11,444
Water/ions	35,201(28)	23,372(36)	23,379(28)
Ligand atoms	–	88	88(S7P), 4 (Zn^2+^)
Total atoms	117,075	81,684	81,701
Minimization (Steep)	50,000	50,000	50,000
NVT equilibration	100 ps	100 ps	100 ps
NPT equilibration	100 ps	100 ps	100 ps
Time steps (fs)	2	2	2
No. of steps	500,000	500,000	500,000
Simulation (ns)	1	1	1

#### Dynamics simulation on *Mtb*GmhA tetramer in absence of ligand

**Figure 7:**
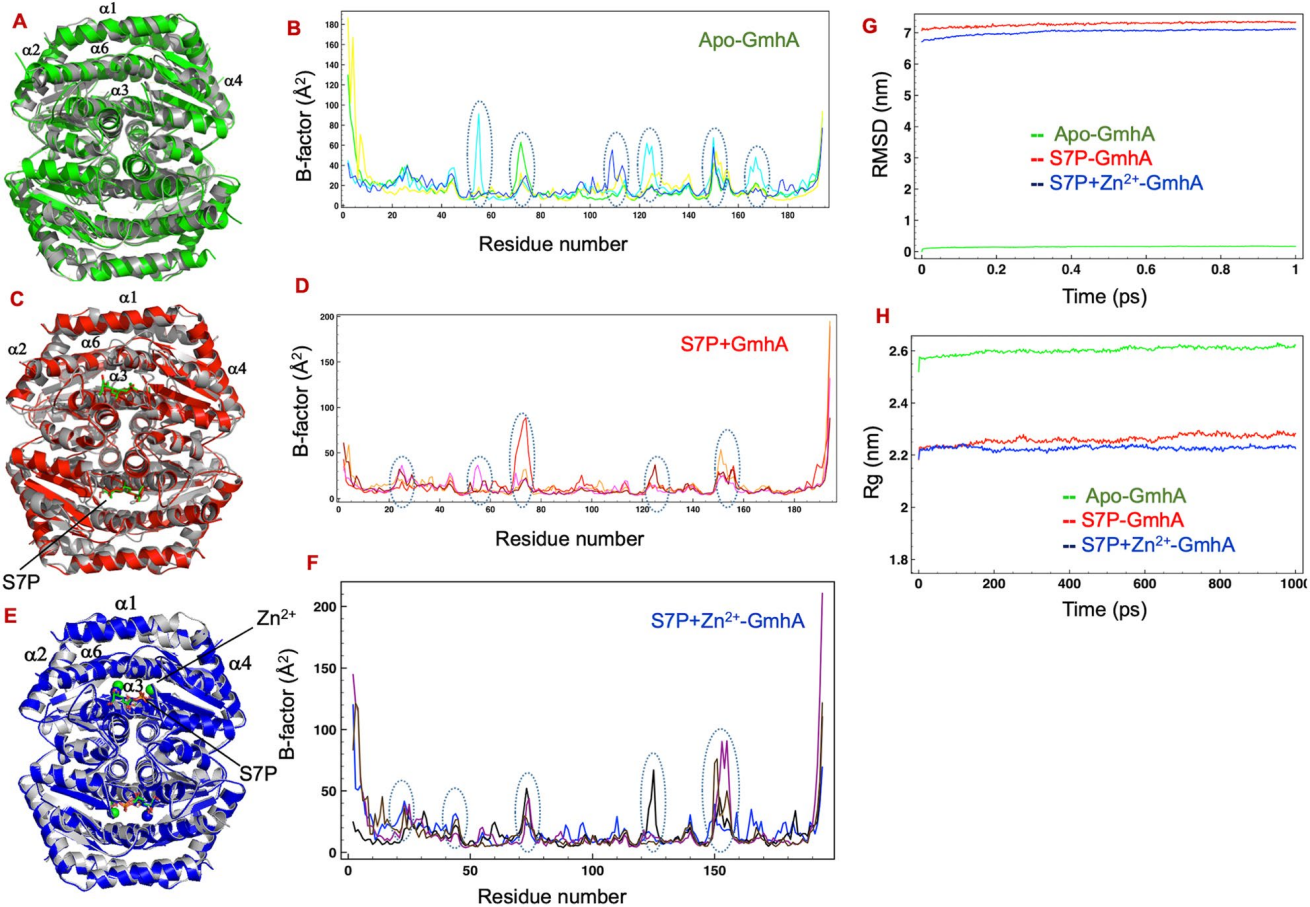
(**A**) Dynamic simulation on apo *Mtb*GmhA tetramer. The simulated *Mtb*GmhA tetramer (Green) is superposed on starting structure (Grey). (**B**) Plot showing the B-factor (Y-axis) and residue number (X-axis) of simulated *Mtb*GmhA tetramer. (**C**) Dynamic simulation on *Mtb*GmhA + D-sedoheptulose 7-phosphate tetramer. The simulated *Mtb*GmhA + D-sedoheptulose 7-phosphate tetramer (red) is superposed on starting structure (grey). (**D**) The B-factor (Y-axis) and residue number (X-axis) of simulated *Mtb*GmhA + D-sedoheptulose 7-phosphate tetramer. (**E**) Dynamic simulation on *Mtb*GmhA + D-sedoheptulose 7-phosphate + Zn^2+^ tetramer. The simulated complex (blue) is superposed on starting structure (grey). (**F**) The B-factor (Y-axis) and residue number (X-axis) of simulated *Mtb*GmhA + D-sedoheptulose 7-phosphate + Zn^2+^ tetramer. (**G**) The plot showing the backbone RMSD ~ time for apo, D-sedoheptulose 7-phosphate bound and D-sedoheptulose 7-phosphate + Zn^2+^ bound *Mtb*GmhA tetramer. (**H**) The plot showing the radius of gyration (R_g_) ~ time, which showed the changes in degree of compactness of *Mtb*GmhA tetramer after D-sedoheptulose 7-phosphate and Zn^2+^ binding.

To investigate the effect of ligand binding on dynamics fluctuation of *Mtb*GmhA tetramer, we have performed the dynamics simulation on *Mtb*GmhA tetramer in absence of ligand. Superposition of simulated *MtbGmhA* tetramer (Green) on starting structure (Grey) have yielded RMSD ~ 1.3 Å for 705 Cα atoms, indicating quite similar structure, except minor changes were observed in the loop regions of protein (Fig. 7A). High B-factors (~ 40–60 Å^2^) were observed for amino acid stretches, 20–30, 50–60, 70–80, 105–115, 120–130, 150–155 and 160–170 of *Mtb*GmhA tetramer (Fig. 7B). The initial RMSD of *Mtb*GmhA tetramer was ~ 0.0 and increased slightly (~ 0.2 nm) during 1 ns simulation (Fig. 7G). The radius of gyration (R_g_) in *Mtb*GmhA tetramer was quite stable during simulation period and found ~ 2.6 nm (Fig. 7H).

#### Dynamics simulation on *Mtb*GmhA tetramer in complex with D-sedoheptulose 7-phosphate

1 ns dynamics simulation was performed on *Mtb*GmhA + D-sedoheptulose 7-phosphate tetramer to analyze the effect of substrate binding on *Mtb*GmhA structure. Superposition of the simulated *Mtb*GmhA + D-sedoheptulose 7-phosphate structure (red) on starting complex structure (grey) have yielded the RMSD ~ 1.1 Å for 740 Cα atoms (Fig. 7C). It showed that overall structure of *Mtb*GmhA tetramer remains unperturbed after dynamics simulation, except minor changes in the loop regions of enzyme. The D-sedoheptulose 7-phosphate substrate was quite stable during simulation period. To examine, whether structural deviations occurred in local regions, or throughout the whole structure, we computed the B-factor plot for *Mtb*GmhA tetramer during entire simulation period (Fig. 7D). The amino acid stretches 20–30, 70–80 and 150–155 showed the higher B-factor (~ 40–60 Å^2^) (Fig. 7D). The *Mtb*GmhA residues *e.g.,* Asn53, Gly55 residues involved in C5-O5 binding and Ser121, Thr122, Ser123, Ser126 residues are involved in C7-PO_4_ binding have shown significant decrease in B factor compared to wild-type *Mtb*GmhA tetramer. The active site Glu66 and Gln173 residues of *Mtb*GmhA showed low B-factor < 20 Å^2^ compared to 20–60 Å^2^ observed in wild type enzyme. These residues bind to C1–O1 and C2–O2 of D-sedoheptulose 7-phosphate and involved in catalysis. Other residues, 20–30, 70–80 and 150–155 showed higher B-factors, though not involved directly in substrate binding.

Figure 7G showed the RMSD of *Mtb*GmhA sampled through dynamics simulation from starting structure. Minor structural changes were observed in 1 ns simulation and reached to final ~ 7 Å. The radius of gyration (R_g_) of complex was quite stable and found ~ 2.2 nm (Fig. 7H). These values were quite similar to R_g_ of *Mtb*GmhA tetramer obtained in small angle X-ray scattering analysis (R_g_ ~ 2.38 nm). These data showed that D-sedoheptulose 7-phosphate binding to *Mtb*GmhA tetramer enhanced the overall stability of enzyme.

#### Dynamics simulation on *Mtb*GmhA tetramer in complex with D-sedoheptulose 7-phosphate and Zn^2+^

Dynamics simulation on *Mtb*GmhA + D-sedoheptulose 7-phosphate + Zn^2+^ tetramer was performed to examine the combined effect of Zn^2+^ and D-sedoheptulose 7-phosphate binding to *Mtb*GmhA. Figure 7E showed the superposition of simulated *Mtb*GmhA + D-sedoheptulose 7-phosphate + Zn^2+^ (Blue) on starting complex structure (Grey) (RMSD ~ 0.5 Å for 752 Cα atoms). Overall structure of *Mtb*GmhA tetramer was quite stable except minor difference in the loop region of the enzyme. The D-sedoheptulose 7-phosphate and Zn^2+^ ions were quite stable in four subunits of *Mtb*GmhA tetramer, except minor movement of Zn^2+^ ions occurred in different subunits. It appears that Zn^2+^ binding further enhanced the stability of *Mtb*GmhA + D-sedoheptulose 7-phosphate tetramer and essential for D-sedoheptulose 7-phosphate binding by *Mtb*GmhA enzyme.

We have computed the B-factor plot for *Mtb*GmhA + D-sedoheptulose 7-phosphate + Zn^2+^ tetramer during the entire simulation period (Fig. 7F). The amino acid stretches, 20–30, 70–80, 120–130 and 150–160 showed the higher B-factor (~ 40–60 Å^2^) (Fig. 7D). The RMSD increased slightly till 20 ps and remained stable throughout 1 ns simulation (~ 0.2 Å) (Fig. 7G). The radius of gyration (R_g_) was quite stable and found ~ 2.2 nm (Fig. 7H).

## Conclusion

In current study, we have performed the structural and biochemical analysis of *Mtb*GmhA enzyme involved in D-α-D-heptose biosynthetic pathway, critical for the development of novel antibiotics against *M. tuberculosis*. The *Mtb*GmhA forms a tetramer in solution and small angle X-ray scattering analysis also yielded the tetrameric envelope of the enzyme. The *Mtb*GmhA catalyzes the D-sedoheptulose 7-phosphate isomerization with 1.45 mM^−1^ s^−1^ rate and binds the *Mtb*HddA and *Mtb*GmhB enzymes in µM range. Site directed mutagenesis have identified the roles of various active site residues involved in D-sedoheptulose 7-phosphate binding and catalysis.

The circular dichroism analysis on wild type and six *Mtb*GmhA mutants showed quite similar secondary structures and high thermostability of the enzyme. The small angle X-ray scattering analysis have revealed de novo SAXS envelope, which fitted well with modeled *Mtb*GmhA tetramer in closed conformation. Dynamics simulation on Apo, D-sedoheptulose 7-phosphate bound and D-sedoheptulose 7-phosphate + Zn^2+^ bound *Mtb*GmhA tetramer showed that ligand binding enhanced the overall stability of the enzyme. In *Mtb*GmhA tetramer, asymmetric domain movement occurred, compared to isolated monomer. The D-sedoheptulose 7-phosphate binding restricts the domain movement and kept the enzyme in the active conformation. The small angle X-ray scattering analysis indicated that *Mtb*GmhA adopts a compact globular conformation, usually observed in crystal structure. This compact conformation of *Mtb*GmhA is catalytically relevant, not only for isomerization of D-sedoheptulose 7-phosphate, but also in binding to other enzymes involved in D-α-D-heptose biosynthesis pathway. Our structural and biochemical analysis on *Mtb*GmhA have provided a new insight into mechanism, which will be critical for novel antibiotics development against *M. tuberculosis*. As *Mtb*GmhA is required to maintain the permeability in *M. tuberculosis*, current knowledge can be implied in other Gram-positive organisms.

## Materials and methods

### Expression and purification

The *Mtb*GmhA gene (*Rv0113*) was amplified from *H37Rv* genomic DNA by polymerase chain reaction and cloned into *pET28a* expression vector (Novagen) using *NdeI* and *HindIII* restriction sites. The *pET28a-Mtb*GmhA plasmid was confirmed by restriction-digestion and gene sequencing analysis. The *pET28a*-*Mtb*GmhA plasmid was transformed in *E. coli BL21(DE3)* cells and cells were grown at 37 °C in luria bertani media (50 µg/ml kanamycin as antibiotic) till OD_600_ ~ 0.6–0.7. The cell culture was induced with 0.5 mM isopropyl β-D-1-thiogalactopyranoside and grown for another 4 h. The *Mtb*GmhA protein overexpressed in soluble fraction of the cell.

The cells were harvested by centrifugation at 10,000 × *g* for 10 min at 4 °C and suspended in lysis buffer containing (25 mM Tris/HCl pH 8.0, 500 mM NaCl, 10% (v/v) Glycerol, 3 mM β-mercaptoethanol, 1 mM Phenylmethylsulfonyl fluoride, 1 mM Benzamidine hydrochloride, 10 mM Imidazole and 0.2 mg/ml Lysozyme). The cells were homogenized, disrupted by sonication and lysate was centrifuged at 25,000 × g to collect the supernatant. The supernatant was loaded on Ni–NTA column (*GE Healthcare*), pre-equilibrated with buffer-A (25 mM Tris/HCl pH 8.0, 300 mM NaCl, 10% Glycerol, 3 mM β-mercaptoethanol, 1 mM Phenylmethyl sulfonyl fluoride and 1 mM Benzamidine hydrochloride). The column was washed with 0–25 mM gradient of imidazole and eluted the protein in buffer-A + 250 mM imidazole. The eluted protein was concentrated and loaded on Superdex 200 column (*GE healthcare Ltd*) pre-equilibrated with buffer (50 mM Tris–HCl pH 8.0, 150 mM NaCl, 10% Glycerol and 3 mM β-mercaptoethanol). The peak fractions were pooled and concentrated using 10 kDa cutoff ultracentrifugal device (*Millipore, USA*). The purified *Mtb*GmhA was analyzed on 12% SDS-PAGE and mass spectrometry. Protein concentration was determined using absorbance at 280 nm and stored at − 80 °C. The recombinant *Mtb*GmhA contains 216 residues e.g. 6 residues from 6xHis tag and 14 residues from vector at N-terminal and 196 residues of *Mtb*GmhA enzyme.

QuickChange II XL site directed mutagenesis protocol (*Stratagene)* was used to generate the six mutants of *Mtb*GmhA. All mutants were purified with protocol used for wild type *Mtb*GmhA purification^21^. All *Mtb*GmhA mutants were confirmed by gene sequencing.

### Small-angle X-ray scattering analysis

The small angle X-ray scattering data was collected by using SAXSpace instrument (Anton Paar, IMTECH, India) at 10 °C using line collimation optics. The *Mtb*GmhA enzyme (conc. ~ 1.2 mg/ml in buffer ~ 25 mM Tris–HCl pH 8.0, 150 mM NaCl and 3 mM β-mercaptoethanol) was used for small angle X-ray scattering data collection. The small angle X-ray scattering data on Lysozyme (conc. ~ 5.0 and 3.4 mg/ml in buffer ~ 40 mM sodium acetate buffer pH 3.8, 150 mM NaCl) was collected for beam intensity estimation and comparison with *Mtb*GmhA data. After data collection, the *Mtb*GmhA enzyme was checked on SDS-PAGE analysis to see if any degradation occurred during data collection. SAXStreat software was used for beam position correction. 120 µl of *Mtb*GmhA was loaded in quartz capillary flow cell and exposed to X-ray beam (λ = 1.5418 Å).

The small angle X-ray scattering data was collected in triplicate and averaged. SAXSquant 4.2.4 software was used to remove the buffer contribution and desmearing the line collimation. The small angle X-ray scattering data were processed using ATSAS 2.8.3 suite and ScÅtter^22^ and data containing scattering intensity (*I*) as a function of scattering vector Q, [where Q = (4π/λ) sinθ, θ is the scattering angle and λ is wavelength] were obtained. The Kratky plot [I(Q)Q^2^ vs Q] was obtained from intensity profile. The radius of gyration (R_g_) and radius of cross section (R_c_) were estimated from scattering intensity at low Q region using Guinier analysis. Additionally, using R_G_ and R_C_ values, the length of an ellipsoidal structure L were estimated using the L = [12(R_G_^2^ − R_C_^2^)] ^½^ relationship. The GNOM45 program was used to estimate the shape and size of scattering entities, which consider both low and high Q data. Indirect Fourier transformation of scattering data over measured Q range was computed as a pairwise distribution function of interatomic vectors^23^. This analysis also provided R_G_ and I_o_ from the second moment and start of P(R) as well as D_max_ (maximum diameter).

Ten independent models of *Mtb*GmhA were generated using DAMMIF program v. 1.1.2^24^, averaged the aligned model and filtered at a given cutoff volume using DAMAVER v. 5.0 program^25^. The *Mtb*GmhA tetramer was superimposed on SAXS envelope using the SUPCOMB v. 2.3 program^26^. The SAXS curve was calculated from the atomic model using the CRYSOL v. 2.8.3 program^27^. The PyMOL v. 2.3 program^28^ was used for structural visualization and to generate all superimposed structures. The curve fitting and data plotting were performed using Graphpad-Prism 5 program^29^.

### Enzyme assay

The activity of wild type and six mutants of *Mtb*GmhA were determined by coupling reaction using *Mtb*HddA, *Mtb*HddA and *Mtb*GmhB enzymes and release of P_i_ was monitored (as described in DeLeon *et. al.*^30^). We prepared the 200 µl of reaction mixture containing (0.31 nM *Mtb*GmhA, 0.11 nM *Mtb*HddA and 0.4 nM of *Mtb*GmhB) in 20 mM HEPES buffer pH 8.0, 10 mM MgCl_2_, 10 mM KCl. 10 mM ATP and 0–3 mM D-sedoheptulose 7-phosphate were added in the reaction mixture and incubated at room temperature for 30 min. The P_i_ concentration was measured using 50 μl of P_i_ colorlock Gold and 0.5 μl of Accelerator (*Innova Biosciences Ltd*) in the reaction mixture. After 2 min, 20 μl of stabilization reagent was added in each sample and absorbance was taken at 630 nm. All reactions were performed in triplicate and 11 different concentrations of D-sedoheptulose 7-phosphate were used for each reaction. The kinetic parameters were determined by fitting the data to Michaelis–Menten equation using GraphPad Prism version 6.0.2 (GraphPad Sofware, La Jolla).

$$v = \frac{{k_{cat} *E_{t} \left[ S \right] }}{{\left( {K_{m} + \left[ S \right]} \right)}}$$

### Binding analysis

The D-sedoheptulose 7-phosphate binding to *Mtb*GmhA was performed using Autolab Esprit Surface Plasmon Resonance equipment^31^. The gold surface [*self-assembled monolayer of 11-mercaptoundecanoic acid (11-MUA)*] was first activated by EDC (N-ethyl-N dimethyl amino propyl carbodiimide, conc. ~ 0.2 M)—NHS (N-hydroxysuccinimide, conc ~ 0.05 M) coupling. 50 µM of *Mtb*GmhA was immobilized on gold surface using 20 mM sodium acetate buffer, pH 4.2. After immobilization, the surface was blocked with 100 mM ethanolamine pH 8.5. The binding experiment was performed at 25 °C in running buffer (20 mM HEPES pH 7.5, 150 mM NaCl, 5 mM MgCl_2_, and 2% Glycerol). The D-sedoheptulose 7-phosphate substrate was injected at 30 µl/min for 5 min, followed by dissociation for 5 min. After each experiment, the sensor surface was regenerated using 50 mM NaOH.

For interaction analysis between *Mtb*GmhA, *Mtb*HddA and *Mtb*GmhB, 50 µM of *Mtb*GmhB was immobilized on gold surface activated by EDC-NHS coupling using 20 mM sodium acetate buffer, pH 4.2. The surface was blocked with 100 mM Ethanolamine pH 8.5. 50 µM of *Mtb*GmhA was injected at 30 µl/min for 5 min followed by dissociation for 5 min. Similar protocol was used for interaction analysis between *Mtb*HddA and *Mtb*GmhB and *Mtb*GmhA and *Mtb*HddA enzymes. All experiments were performed at 25 °C. The sensograms were collected, processed, analyzed using BIAevaluation (GE Healthcare)^32^ and fitted with Langmuir 1:1 model using formula

$$R_{eq} = \frac{{R_{max} *K_{A } * C_{A} }}{{K_{A} *C_{A} + 1}}$$

where R_eq_ is binding response at steady state, R_max_ is maximal binding response, C_A_ is the concentration of analyte and K_A_ is equilibrium association constant.

### Circular dichroism analysis

Circular dichroism analysis was performed to estimate the secondary structure of *Mtb*GmhA enzyme^33^. A far UV spectrum on *Mtb*GmhA was collected using Chirascan spectropolarimeter (Applied Photophysics) with quartz cuvette of 1 mm path length. The *Mtb*GmhA enzyme was transferred in Sodium phosphate buffer, pH 7.5. The circular dichroism data was collected in 200–260 nm range at 25 °C. The spectra from baseline was subtracted from protein spectra and obtained values were averaged for each dataset. The mean residue ellipticity (θ) was calculated from the observed spectra, as a function of wavelength. The K2D2 program was used to estimate the secondary structure of *Mtb*GmhA^34^. Similar protocol was used for CD data collection on all six *Mtb*GmhA mutants. The theoretical secondary structure prediction on *Mtb*GmhA was performed using various programs e.g., SOPMA^35^, CFSSP^36^, GOR^37^, PHD^38^, SIMPA96^39^, DSC^40^, HNN^41^, RaptorX^42^, JPred^43^ and PsiPred^44^.

For thermal stability analysis, the circular dichroism spectra of *Mtb*GmhA was recorded at 222 nm from 25 to 90 °C range in 10 °C step. The melting temperature was calculated from scattering profile using polynomial fitting, taking temperature corresponding to half denaturation^45^. A graph of (ΔA/ΔT) versus (T_avg_) was plotted using Sigma Plot program. A bell-shaped melting curve was observed, and peak corresponds to melting temperature of the protein.

### Multiple sequence alignment

The *Mtb*GmhA sequence was retrieved from UniProt (P9WGG1) database and sequences of 11 GmhA orthologues were retrieved from RCSB database^46^ The *Mtb*GmhA sequence was aligned with 11 GmhA orthologues using MultAlin^47^ and ESPript 3.0^48^ programs.

### Molecular modeling, docking and dynamics simulation

I-TASSER server was used to model the *Mtb*GmhA tetramer and was ranked by the C-score and TM-score^49^. The *Mtb*GmhA sequence (1–196 residues) was given as input and server retrieved the template proteins of similar folds from Protein Data Bank by LOMETS (Local Meta-Threading-Server)^50^. Additional steps were performed to remove the steric clashes and refined the global topology of the *Mtb*GmhA models.

I-TASSER server identified the PDB-2X3Y (Crystal structure of GmhA tetramer in closed state from *B. pseudomallei*^12^) as the best template for *Mtb*GmhA modeling (RMSD = 0.79, id1 = 0.39, id2 = 0.39, Conv = 0.98, Z-score = 3.2). The best *Mtb*GmhA model was obtained having following parameters (C-score = 0.21 and TM-score = 0.88 ± 0.07), which indicated a reliable model with correct global topology. The I-TASSER server also yielded PDB-2I2W (Crystal structure of *Escherichia Coli* Phosphoheptose Isomerase in open state^10^) as another template for *Mtb*GmhA modeling, however following parameters (RMSD = 2.1, TM id1 = 0.35, TM-score = 0.82, Conv = 0.89) did not warrant a reliable *Mtb*GmhA model in “open state”.

Docking of D-sedoheptulose 7-phosphate and Zn^2+^ into *Mtb*GmhA tetramer was performed using GLIDE module of the SchroeÖdinger-9.4 program^51^. The *Mtb*GmhA + S7P + Zn^2+^ complex was generated using induced fit protocol of SchrÖdinger-9.4 program. Defaults parameters were used except Extra Precision (EP) scoring function was used in docking calculation.

The dynamics simulation of (i) Apo (ii) D-sedoheptulose 7-phosphate bound and (iii) D-sedoheptulose 7-phosphate + Zn^2+^ bound models of *Mtb*GmhA tetramer were performed using GROMACS package 4.5 (GROningen MAchine for Chemical Simulations) using CHARMM all-atom force field parameters (Table 4)^52^. The *Mtb*GmhA tetramer was solvated in the dodecahedron box at 1 nm distance from the boundary. Simple point charge water molecules were used for solvation together with Na^+^ and Cl^−^ ions to neutralize the total charge of the system. Energy minimization was performed using 50,000 steps of steepest descent minimization. To maintain the 300 K constant temperature, protein and non-protein atoms were coupled to their temperature baths using V-scale thermostat using 100 ps NVT. The whole system was kept for 100 ps NPT equilibration and 1 bar pressure. For all simulations, the bond lengths and water molecules were restrained using the SETTLE and LINCS^53^ algorithms respectively.

The 1 nm cutoff was used for the treatment of Van der Waals interactions and long-range electrostatic interactions and simulated using PME^54^ method with 0.16 FF grid spacing and 4^th^ order B-spline interpolation for the reciprocal sum space. Periodic boundary conditions were applied in all directions. Rigid body displacements and rotations were removed from all trajectories. The PRODRG server^55^ was used to generate the topology of the D-sedoheptulose 7-phosphate. PyMol and Plot2^56^ programs were used for generating the figures and all simulation trajectories. The *Mtb*GmhA tetramers obtained after dynamics simulation exhibited good stereochemistry. The structural validation on all models was performed using various servers *e.g.,* ERRAT^57^, Verify3D^58^, and PROCHECK^59^.

## Supplementary information

Supplementary information.
