# Supplementary material for: Structural and functional characterization of M. tuberculosis sedoheptulose- 7-phosphate isomerase, a critical enzyme involved in lipopolysaccharide biosynthetic pathway
Source: Sci Rep. 2020 Nov 30;10:20813. doi: 10.1038/s41598-020-77230-8 (PMC7705670; doi:10.1038/s41598-020-77230-8)

Structural and functional characterization of *M. tuberculosis* Sedoheptulose-7-Phosphate Isomerase, a critical enzyme involved in lipopolysaccharide biosynthetic pathway

Sumita karan[1], Bhanu Pratap[1], Shiv Pratap Yadav[2], FNU Ashish[2] and Ajay K. Saxena[1]#

Table S1. Primers used in gene amplification of six *Mtb*GmhA mutants. Nucleotides in the bold letter showed the position of mutation. Underlined regions indicate codon that will be altered after mutagenesis.

Mutation	Mutagenesis Primer (5’-3’)
N53G forward	5’GTCTTCATGTGTGGC**GG**CGGTGGTAGCGCTGCG3’
N53G reverse	5’ CGCAGCGCTACCACCG**CC**GCCACACATGAAGAC3’
S121G forward	5’ ACGCTTTTTGCGATA**G**GTACCTCCGGCAATTCT3’
S121G reverse	5’AGAATTGCCGGAGGTAC**C**TATCGCAAAAAGCGT3’
T122A forward	5’CTTTTTGCGATAAGT**G**CCTCCGGCAATTCTATG 3’
T122A reverse	5’CATAGAATTGCCGGAGG**C**ACTTATCGCAAAAAG3’
S123A forward	5’ TTTGCGATAAGTACC**G**CCGGCAATTCTATGAGT3’
S123A reverse	5’ACTCATAGAATTGCCGG**C**GGTACTTATCGCAAA3’
S126A forward	5’AGTACCTCCGGCAAT**G**CTATGAGTGTACTGCGG3’
S126A reverse	5’CCGCAGTACACTCATAG**C**ATTGCCGGAGGTACT3’
Q173A forward	5’GACACCGGGCGAATC**GC**GGAATCTCACATCGTT 3’
Q173A reverse	5’AACGATGTGAGATTCC**GC**GATTCGCCCGGTGTC3’

Table S2. Secondary structural contents in *Mtb*GmhA enzyme obtained from various theoretical structural prediction programs.

**Techniques**	**α–helix**	**β–sheet**	**random coil**
RAPTOR	51%	12%	35%
SOPMA	54%	14%	31%
GOR1	57%	32%	11%
PHD	57%	14%	29%
SIMPA96	49%	13%	37%
DSC	57%	8%	35%
HNN	46%	16%	38%
PRED	48%	14%	38%
DPM	48%	14%	38%

Fig. S1. SDS-PAGE analysis of purified *Mtb*GmhA enzyme eluted from Superdex 200 column. M- molecular weight marker, E1 to E9 represents the 9 eluted fractions of *Mtb*GmhA enzyme.

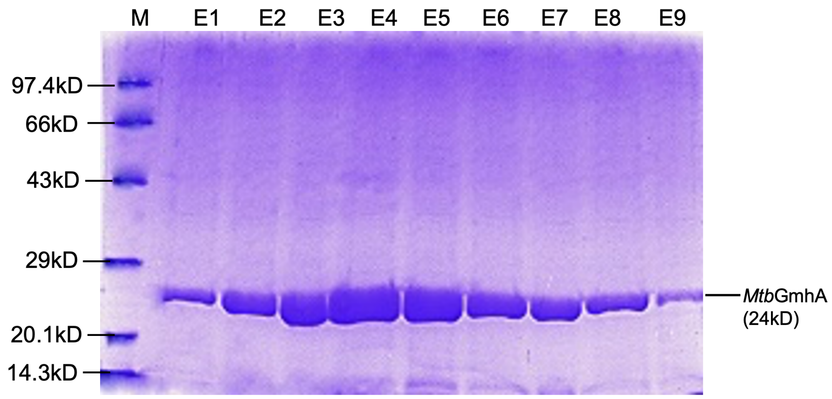

Fig. S2. Bar diagram showing the catalytic efficiency of wild type and six *Mtb*GmhA mutants.

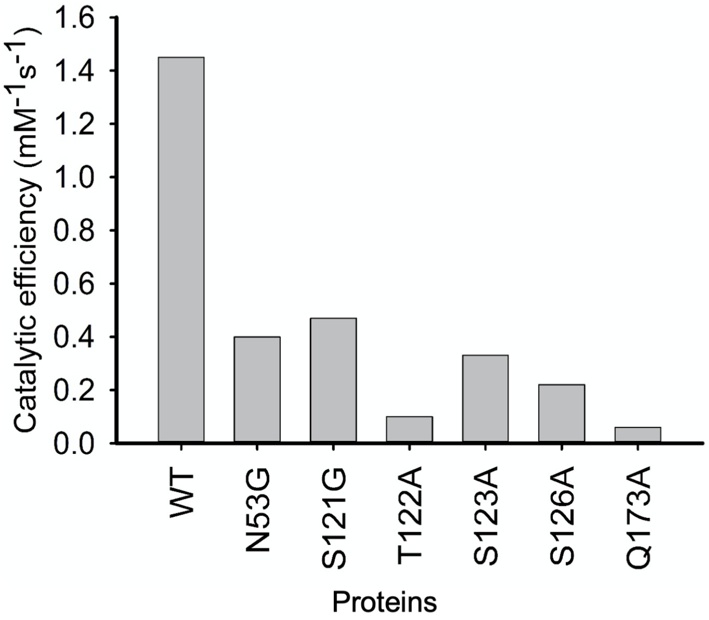

Fig. S3. Validation of stereochemical properties of Apo *Mtb*GmhA tetramer obtained after dynamics simulation. **(A)** Ramachandran Plot shows most of the residues lie in allowed regions of the plot. **(B)** ProSA-web analysis showed Z score = – 6.1, indicating the overall good quality of the simulated model. **(C-F)** ERRAT plot of four *Mtb*GmhA monomers showing the regions in structure that can be rejected at the 95% confidence limit (yellow). 5% of a good protein structure is expected to have an error value above this level. **(G)** Verify 3D plot showing the threshold score > 0.2 for *Mtb*GmhA tetramer.

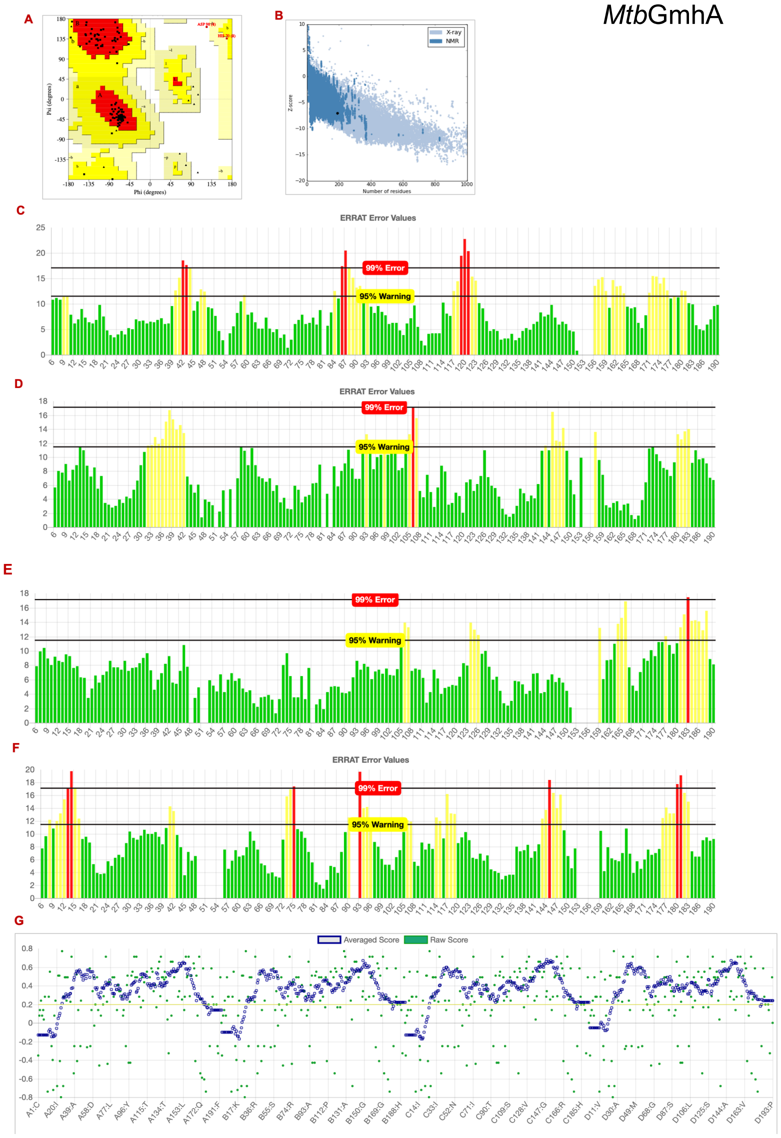

Fig. S4. Validation of stereochemical properties of *Mtb*GmhA+S7P tetramer obtained after dynamics simulation. **(A)** Ramachandran Plot shows most of the residues lie in allowed regions of the plot. **(B)** ProSA analysis showed Z score= – 6.0, indicating the overall good quality of the simulated model. **(C-F)** ERRAT plot of four *Mtb*GmhA monomers showing the regions in structure that can be rejected at the 95% confidence limit (yellow). 5% of a good protein structure is expected to have an error value above this level. **(G)** Verify 3D plot showing the threshold score > 0.2 for *Mtb*GmhA tetramer.

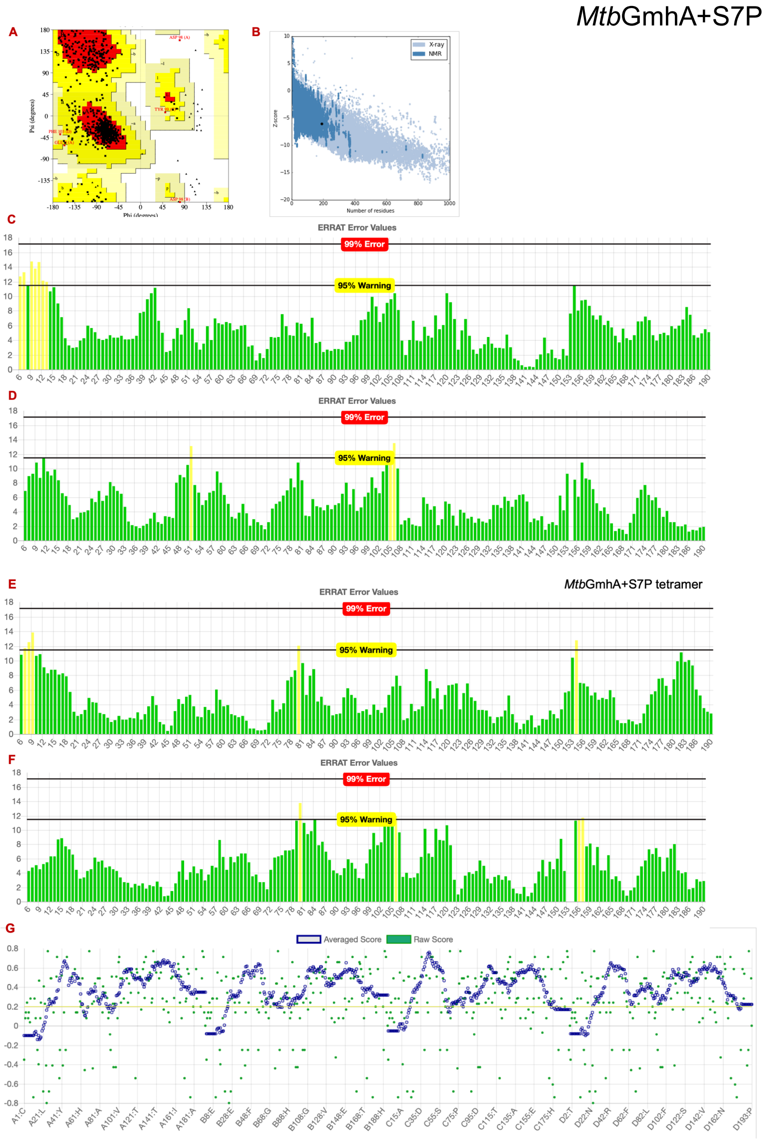

Fig. S5. Validation of stereochemical properties of *Mtb*GmhA+S7P+Zn^{2+} tetramer obtained after dynamics simulation. **(A)** Ramachandran Plot shows most of the residues lie in allowed regions of the plot. **(B)** ProSA analysis showed Z score = – 6.2, indicating the overall good quality of the simulated model. **(C-F)** ERRAT plot of four *Mtb*GmhA monomers showing the regions in structure that can be rejected at the 95% confidence limit (yellow). 5% of a good protein structure is expected to have an error value above this level. **(G)** Verify 3D plot showing the threshold score > 0.2 for *Mtb*GmhA tetramer.

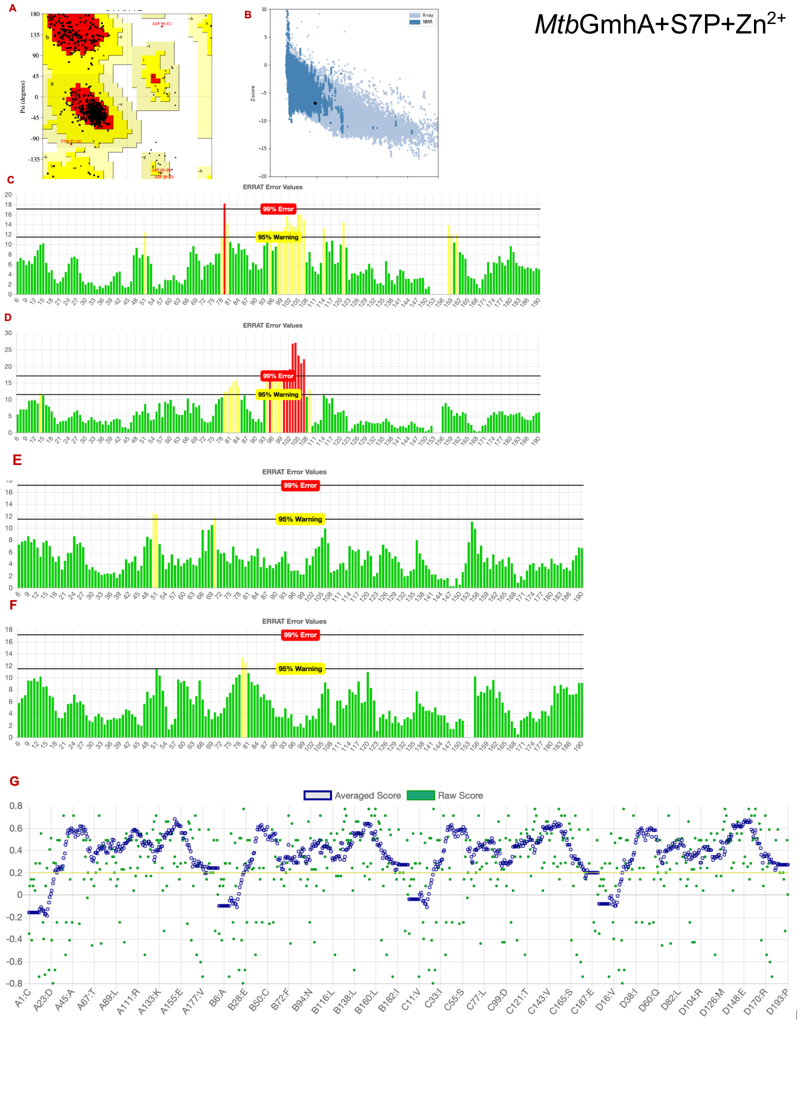

Supplement: Supplementary file 1 — Supplementary information. [file 41598_2020_77230_MOESM1_ESM.pdf]
